# Marine Lipids on Cardiovascular Diseases and Other Chronic Diseases Induced by Diet: An Insight Provided by Proteomics and Lipidomics

**DOI:** 10.3390/md15080258

**Published:** 2017-08-18

**Authors:** Lucía Méndez, Gabriel Dasilva, Nùria Taltavull, Marta Romeu, Isabel Medina

**Affiliations:** 1 Instituto de Investigaciones Marinas (IIM-CSIC), Eduardo Cabello 6, E-36208 Vigo, Spain; gabrielsilva@iim.csic.es (G.D.); medina@iim.csic.es (I.M.); 2 Unitat de Farmacologia, Facultat de Medicina i Ciències de la Salut, Universitat Rovira i Virgili, Sant Llorenç 21, E-43201 Reus, Spain; nuria.taltavull@urv.cat (N.T.); marta.romeu@urv.cat (M.R.)

**Keywords:** marine lipids, EPA, DHA, proteomics, lipidomics, metabolic syndrome, cardiovascular disease, type 2 diabetes

## Abstract

Marine lipids, especially ω-3 polyunsaturated fatty acids (PUFAs) eicosapentaenoic acid (EPA) and docosahexaenoic acid (DHA), have largely been linked to prevention of diet-induced diseases. The anti-inflammatory and hypolipidemic properties of EPA and DHA supplementation have been well-described. However, there is still a significant lack of information about their particular mechanism of action. Furthermore, repeated meta-analyses have not shown conclusive results in support of their beneficial health effects. Modern “omics” approaches, namely proteomics and lipidomics, have made it possible to identify some of the mechanisms behind the benefits of marine lipids in the metabolic syndrome and related diseases, i.e., cardiovascular diseases and type 2 diabetes. Although until now their use has been scarce, these “omics” have brought new insights in this area of nutrition research. The purpose of the present review is to comprehensively show the research articles currently available in the literature which have specifically applied proteomics, lipidomics or both approaches to investigate the role of marine lipids intake in the prevention or palliation of these chronic pathologies related to diet. The methodology adopted, the class of marine lipids examined, the diet-related disease studied, and the main findings obtained in each investigation will be reviewed.

## 1. Introduction

### 1.1. Chronic Diseases Induced by Diet: A World Health Problem

The intake of westernized diets, which are rich in refined carbohydrates, cholesterol, saturated and trans fats, and have an increased ratio of ω-6/ω-3 PUFAs, along with a sedentary lifestyle can quickly lead to the development of different pathologies and metabolic disorders [1]. These chronic diseases related to diet, such as cardiovascular diseases (CVD), obesity, overweight, hypertension, type 2 diabetes, hyperlipidemia, hyperinsulinemia, osteoporosis, osteopenia or cancer, are considered the epidemic of modern societies. It is estimated that 20–30% of the adult population in these countries suffers Metabolic Syndrome (MetS) [2]. The MetS is a compilation of risk factors associated with CVD and type 2 diabetes. These factors include dyslipidemia (decreased high-density lipoprotein (HDL) cholesterol levels and increased low-density lipoprotein (LDL) cholesterol and triglycerides levels), hyperglycemia, hypertension, insulin resistance and obesity. Inflammation and oxidative stress have also been closely related to MetS and derived diseases [3].

According to the Eurostat report of May 2016, the first two leading causes of death in Europe were CVD and cancer, well above respiratory diseases, which were the third most common cause of death [4]. The average in Europe was 132 deaths/100,000 inhabitants due to CVD in 2013, being 350/100,000 in countries like Hungary or Slovakia, and less than 100/100,000 in Spain, Portugal or Greece. Data from the World Health Organization (WHO) in 2015 also showed that the leading causes of death in high-income countries were CVD and cancer, while infectious diseases were the leading cause of death in low-income countries [5].

Therefore, the study of diet components and their relation to the progression of chronic diseases and metabolic disorders has arisen as a field of great interest for the scientific community. Accordingly, the number of publications found in the Scopus database (www.scopus.com) which are focused on this topic has exponentially grown, especially since 1990. Considering the search criteria: TITLE-ABS-KEY (nutrition OR diet) AND TITLE-ABS-KEY (“metabolic disorder” OR “metabolic alteration” OR obesity OR “metabolic syndrome” OR diabetes OR “cardiovascular disease” OR cholesterol OR insulin OR atherosclerosis OR inflammation OR “oxidative stress”), around 272,000 were found. From these, 75% are research articles and 14% are reviews. The rest of the published work consists of book chapters, notes, conferences, etc. In relation to the field of study, 76% of the publications belong to the field of medicine, 27% to biochemistry, and the rest of them are framed in the fields of agricultural and biological sciences, nursing, pharmacology, toxicology and pharmaceutics, neuroscience, chemistry, immunology and microbiology, etc.

As a consequence of these investigations, huge quantities of bioactive compounds have been found to exert beneficial effects on human health. Among that, ω-3 PUFAs from marine origin have quickly gained more attention. Currently, there is considerable evidence that the intake of marine-origin polyunsaturated fatty acids (PUFAs), especially eicosapentaenoic acid (EPA) and docosahexaenoic acid (DHA), can help in the prevention/palliation of inflammatory processes and metabolic diseases, although their mechanisms of action are not entirely understood yet.

### 1.2. Marine Lipids as Bioactive Compounds against MetS and Chronic Diseases Induced by Diet

Marine lipids, especially EPA and DHA, have largely demonstrated their bioactivity in human health. The interest in their intake arose from a series of pioneering studies in the Inuit Eskimo population during the 1960s and 1970s. These studies reported a lower incidence of cardiovascular pathologies in that population associated with a high intake of fish in their diet. Later, similar relationships were discovered in human populations from other regions, particularly in Iceland and Alaska Natives [6].

In addition to these investigations, marine ω-3 PUFAs have been shown to alleviate metabolic disorder symptoms, such as heart disease, diabetes, obesity and insulin resistance. Since the late 1950s, the number of publications found in the Scopus database (TITLE-ABS-KEY (“omega 3” OR “PUFA” OR “eicosapentaenoic” OR “docosahexaenoic” OR “marine fatty acids” OR “marine lipid” OR “marine oil” OR “fish oil” OR “fish lipid”) AND TITE-ABS-KEY (nutrition OR diet) AND TITLE-ABS-KEY (“metabolic disorder” OR “metabolic alteration” OR “metabolic disease” OR obesity OR “metabolic syndrome” OR diabetes OR “cardiovascular disease” OR cholesterol OR insulin OR atherosclerosis OR inflammation OR “oxidative stress”)), which have related the consumption of marine fatty acids or lipids to these metabolic disorders, has increased exponentially, mainly since the late 1970s. On the whole, more than 13,100 publications can currently be found in the Scopus database. Sixty-five percent of these publications are research articles, 23% are reviews and the rest of them are book chapters, notes, conferences, etc.

These research articles have used a plethora of different approaches (from chemistry or biochemistry to phycology) and a huge variety of methodologies to address this topic. As a consequence, the publications are categorized in very diverse fields of study, medicine (74%), biochemistry (30%), agricultural and biological sciences (20%), nursing (19%) and the rest of the articles are framed in the fields of pharmacology, toxicology and pharmaceutics, chemistry, neuroscience, immunology and microbiology, phycology, social sciences, mathematics, computer science, etc.

The aim of the present review is to comprehensively show those research articles currently available in the literature which have specifically used proteomics, lipidomics or both approaches to investigate the role of marine lipids intake in the prevention/palliation of chronic pathologies induced by the consumption of westernized diets, namely MetS, CVD and type 2 diabetes. Our ultimate objective is to highlight the usefulness of the application of these approaches to nutrition research, in order to discover the underlying mechanisms of the beneficial effects of marine lipids in human metabolic health, since its translation to practice via nutritional interventions is still in its infancy.

## 2. Omics for Unrevealing Mechanisms: Proteomics and Lipidomics

Almost all cellular processes, from gene expression to synthesis, degradation and protein activity, can be affected by diet and lifestyle. Therefore, nutrients and other food components (including marine lipids) may alter metabolic functions in cells in a quite complex way. This complex relationship between nutrition and health makes nutrition research an ideal field for the application of systems biology approaches [7], as being holistic and integrated. Systems biology encompasses a broad range of functional areas called “omics” These areas include genomics, transcriptomics, proteomics, metabolomics, lipidomics and bioinformatics [8]. This new outlook started as a result of the human genome project in the early 2000s [9].

The number of publications found in the literature which incorporates the most novel omics approaches (proteomics and lipidomics) to the study of the beneficial effects of marine lipids against MetS and their associated chronic diseases is still scarce. Proteomics and lipidomics studies applied to nutrition research have to cope with a variety of difficulties ranging from experimental design, sample processing and optimization strategies to data analysis and identification. The need for high-sensitivity modern mass spectrometers combined with bioinformatics resources increases costs and requires highly qualified staff. In spite of these difficulties, the interest of the scientific community in these approaches has been progressively growing, in parallel with analytical advancements in liquid chromatography, mass spectrometry and bioinformatics.

In the following subsections, we review the research articles currently available in the literature which have used proteomics (Section 2.1), lipidomics (Section 2.2) or both (Section 2.3) to analyze the effects of the consumption of marine lipids on the development and progression of human MetS as well as their associated chronic diseases (CVD and type 2 diabetes).

### 2.1. Beneficial Effects of Marine Lipids Intake Assayed by Proteomics

Proteins are important mediators of biological activities in all living cellular units. Proteomics is the large-scale study of proteins and therefore comprises the methodologies used for the study of a proteome [10]. The “proteome” a term firstly coined by Wilkins et al. in the early 2000s, is comprised of all expressed proteins encoded by the genome of a cellular system, including all cellular proteins and all protein species (isoforms and protein modifications) [11,12].

The study of how ω-3 PUFAs regulate proteins and metabolic pathways may significantly contribute to understanding the putative mechanisms by which marine lipids elicit their beneficial health effects. In fact, nutrition research has lately adopted the proteomics tools to measure changes in the protein complement of a biological system. This adoption has enabled modeling of biological processes in response to dietary marine lipids, as well as the elucidation of novel biomarkers for health or disease which are sensitive to such lipids [13]. There are limited studies which have addressed the influence of marine lipids on proteome with the aim to investigate their effects against human metabolic disorders. These studies have mainly used classical two-dimensional gel electrophoresis (2-DE) combined with mass spectrometry (MS) to elucidate changes in metabolic pathways and target proteins which may explain a certain beneficial effect on human health. This traditional methodology has made it possible to identify changes in several metabolic pathways, such as glucose and fatty acid metabolism, oxidative stress, antioxidant defense mechanisms and redox status. The study of minor abundant proteins such as those participating in inflammatory pathways requires the introduction in nutrition research of more quantitative and sensitive methods, like multiple reaction monitoring (MRM) and multiplexed immunoassays. Both strategies might be useful for the evaluation and validation of newly discovered candidate biomarkers in human biofluids.

By using Scopus database, 53 publications have been found following the search criteria: TITLE-ABS-KEY (“omega 3” OR “PUFA” OR “eicosapentaenoic” OR “docosahexaenoic” OR “marine fatty acids” OR “marine lipid” OR “marine oil” OR “fish oil” OR “fish lipid”) AND TITE-ABS-KEY (nutrition OR diet) AND TITLE-ABS-KEY (“metabolic disorder” OR “metabolic alteration” OR “metabolic disease” OR obesity OR “metabolic syndrome” OR diabetes OR “cardiovascular disease” OR cholesterol OR insulin OR atherosclerosis OR inflammation OR “oxidative stress”) AND TITE-ABS-KEY (proteomic OR proteomics OR proteome). These publications, mainly research articles (56%) and reviews (30%), belong, in descending order of abundance, to the fields of biochemistry, genetics and molecular biology (63%) and medicine (58%), and to a lesser extent nursing, agricultural and biological sciences, pharmacology, toxicology and pharmaceutics, immunology and microbiology, neuroscience, chemistry, etc. 

In this review, only research articles focusing on proteomics are presented. The studies which have used ω-3 PUFAs from a vegetal origin, i.e., α-linolenic acid (ALA), have been excluded. Only articles which have assayed the effect of marine lipids on human metabolic disorders related to diet (i.e., features of MeS, cardiovascular diseases and type 2 diabetes) and those that have used animal or cellular models to mimic these alterations are presented. Moreover, articles which have combined both lipidomics and proteomics tools in the same research are shown jointly in Section 2.3. 

According to these filters, 14 research articles have been found. All of them investigated the effect of EPA or DHA or both on metabolic health. Most of these studies used bottom-up proteomics approaches, based on 2-DE coupled with MS protein identification. Novel gel-free proteomics techniques based on MS, such as shotgun proteomics, have scarcely been used yet, as noted in Table 1.

#### 2.1.1. Proteomics in Clinical Trials

Two studies analyzed the effect of the supplementation of a mix of EPA and DHA on the modulation of the peripheral blood mononuclear cells (PBMCs) proteome after acute intake [14] or after 12 weeks of dietary intervention [15] in patients with MetS. In the first case, proteomics analysis identified five proteins related to cell signaling and interaction, DNA repair, cellular assembly and organization, and cell morphology which were regulated by acute consumption of marine PUFAs. 

In the second study, the prolonged intake of EPA and DHA regulated 17 proteins of PBMCs proteome involved in immunological diseases and inflammatory response, down-regulating proteins directly related to oxidative stress, inflammation, endoplasmic reticulum stress and DNA repair. A third study, which was carried out with the same dietary intervention and proteomics tools, evaluated the changes induced in subcutaneous white adipose tissue (WAT) proteome after 12 weeks of EPA and DHA intake [16]. Three proteins of glucose metabolism were found down-regulated by marine PUFAs supplementation. These proteins were correlated with lower systemic insulin resistance and improved insulin signaling in subcutaneous WAT of the MetS patients.

Also in humans, De Roos et al. [17] studied mechanisms involved in preventing the early onset of coronary heart disease (CHD) through fish oil intake. Authors identified ten serum proteins that were down-regulated in healthy volunteers after the consumption of a daily dose of 3.5 g of fish oil for 6 weeks. These altered serum proteins, metabolically related to lipoprotein metabolism and inflammation, led to a significant shift towards the larger, more cholesterol-rich HDL2 particle which might imply that fish oil activated anti-inflammatory and lipid modulating mechanisms believed to impede the early onset of CHD. This specific effect of fish oil on HDL metabolism has later been investigated by Burillo et al. [18]. They compared the proteome of HDL before and after ω-3 PUFAs intake for 5 weeks. Healthy smoker volunteers ingested a commercial mixture of marine and non-marine ω-3 fatty acids. The consumption of marine ω-3 PUFAs up-regulated seven proteins related to the antioxidant, anti-inflammatory and anti-atherosclerotic properties of HDL. Likewise, marine lipids down-regulated six proteins involved in the regulation of complement activation and acute phase response. Moreover, the modification of lipoprotein containing apoAI (LpAI) proteome suggested that the protein changes found might have improved the functionality of the particle.

#### 2.1.2. Proteomics in Animal Models and Cell Cultures

Besides clinical trials, several studies have used animal or cell models to investigate the role of marine ω-3 PUFAs in regulating cellular proteomes which are rather difficult to analyze in humans, such as the liver proteome. In a study carried out in C57BL/6 mice, Ahmed et al. [19] investigated the regulation of the liver proteome by a dietary intervention with 10% ω-3 PUFAs from menhaden oil for 4 months. Proteomics data showed that fish oil up-regulated eight proteins related to lipid, carbohydrate, protein and one-carbon metabolism and the citric acid cycle, which involved an integrated regulation of metabolic pathways by fish oil. This proteome modulation was correlated with a significant reduction of plasma triglycerides and free fatty acid (FFA) levels. 

Likewise, other studies investigated the effect of fish oil when it was added to high fat, cholesterol and sucrose diets. The first study [20] evaluated changes in the liver mitochondrial subproteome, an organelle which plays a critical role in cell metabolism and the development of metabolic alterations, induced in rats by the supplementation with 10% fish oil of a high fat diet for 50 weeks as compared to rats fed low fat diet. As a result, Wrzesinski et al. identified 54 mitochondrial proteins regulated by fish oil. These proteins were involved in fatty acid oxidation and amino acid metabolism as well as in the increase of oxidative phosphorylation.

In a second research, De Roos et al. [21] studied the modulation of the liver proteome by fish oil supplementation of a diet high in saturated fat and cholesterol (HFC) for 3 weeks in APOE*3 Leiden transgenic mice, a model for lipid metabolism and atherosclerosis. These authors identified up to 44 proteins altered by fish oil as compared to HFC, which were mainly involved in glucose and lipid metabolism, as well as oxidation and aging processes. These changes were correlated with lower plasma and liver cholesterol and triglycerides levels as well as minor plasma FFA and glucose but higher plasma insulin levels, revealing new insights into mechanisms by which these fish oils can regulate lipid metabolism and related pathways.

A third research [22] reported the physiological modulation of rat liver proteome induced by 24-week supplementation with fish oil considering two different background diets: standard or high in fat and sucrose (HFHS). Méndez et al. demonstrated a different capacity of fish oil for regulating liver proteins depending on the background diet (6 proteins into standard diet and 31 into HFHS diet). Proteome changes induced by fish oil, especially under HFHS diet, consisted of decreasing the level of enzymes from lipogenesis and glycolysis, enhancing fatty acid beta-oxidation and insulin signaling and ameliorating endoplasmic reticulum stress. Moreover, the consumption of fish oil decreased protein oxidation and improved several biochemical parameters. 

Other authors have used cell culture experiments to evaluate EPA and DHA effects on the proteome. Kalupahana et al. [23] investigated the effects of EPA on proteins from 3T3-L1 adipocytes treated with either EPA or arachidonic acid (ARA). EPA-treated cells presented higher levels of 19 proteins involved in carbohydrate and fatty acid metabolism and several other proteins related to cellular metabolism including response to stress. Likewise, EPA treatment resulted in lower levels of eight proteins belong to lipogenesis and other cellular metabolic processes such as cytoskeleton organization and biogenesis. 

Proteomics has also been employed to evaluate the differential effect of the main marine ω-3 PUFAs. Mavrommantis et al. [24] analyzed the potentially different effects on the liver proteome regulation exerted by the dietary supplementation either with fish oil (EPA and DHA) or DHA alone in apoE knockout mice (model of atherosclerosis) fed HFC diet for 2 weeks. Both DHA and fish oil regulated 35 liver proteins, mainly involved in the metabolism of lipoproteins and oxidative stress. However, their effects on the proteome were not the same, since four of these proteins were differentially modulated by DHA or fish oil. As a consequence, although both fish oil and DHA could beneficially affect lipoprotein metabolism and oxidative stress, intervention with fish oil but not with DHA resulted in significantly lower levels of hepatic soluble epoxide hydrolase, an enzyme closely related to cardiovascular disease, as compared to control oil. This different behavior between EPA and DHA was also highlighted by Johnson et al. [25]. These authors evaluated the potentially independent protective/reversal effect of dietary EPA or DHA against mitochondrial dysfunction in aging skeletal muscle, after diet supplementation of young or older C57BL/6 mice. Authors found that 10-weeks of dietary supplementation with EPA but not with DHA partially attenuated the age-related decay in mitochondrial function. Thirty-nine mitochondrial proteins changed between old control and old EPA-treated mice. Thirty-two mitochondrial proteins changed between old control and old DHA-treated mice. However, only three proteins for EPA supplementation and seven for DHA were coincident with those proteins which were found to differ in young and old control mice. Authors concluded that neither EPA nor DHA attenuated the age-related drop in mitochondrial protein content, although these changes demonstrated that both EPA and DHA exerted some common biological effects (anticoagulation, anti-inflammatory, reduced FXR/RXR activation). Additionally, proteomics data showed that EPA improved muscle protein quality, specifically by decreasing mitochondrial protein carbamylation, a post-translational protein modification (PTM) that is driven by inflammation.

#### 2.1.3. Proteomics for Studying Post-Translational Protein Modifications (PTMs)

Abundant evidence has shown that ω-3 PUFAs influence redox homeostasis [26] and several researchers have described both antioxidant and pro-oxidant properties for these fatty acids. Since diet-induced metabolic diseases are often associated with increased oxidative stress, the characterization of oxidative PTMs seems to be critical for understanding the mechanisms underlying the action of marine ω-3 PUFAs in theses pathologies. However, studies focused on the effects of EPA and DHA in modulating protein quality, besides protein quantity, are scarce likely due to methodological limitations.

Among the oxidative PTMs, protein carbonyl moieties formed in proteins (protein carbonylation), which are the more common oxidative PTMs, are considered as a major hallmark of oxidative protein damage. Metabolic alterations have strongly been correlated with high levels of protein carbonylation. However, only a very few studies have used proteomics tools to identify protein carbonylation targets in metabolic diseases and evaluate the potential effects that marine lipids exerted on them. A study addressed the effect of various dietary EPA and DHA ratios (1:1, 2:1, and 1:2, respectively) on protein carbonylation from plasma, kidney, skeletal muscle, and liver [27] in rats after 13 weeks of dietary intervention. Rats fed soybean (rich in ω-6 linoleic acid (LA)) or linseed oil (rich in ω-3 ALA) were used as controls. Authors identified targets of protein carbonylation in all the analyzed tissues. The three fish oil ratios, especially the 1:1 EPA:DHA, exerted a selective-protective effect against carbonylation of six proteins from plasma and liver, which was correlated with the improvement of biochemical features.

Jourmard-Cubizolles et al. [28] studied a specific oxidative PTM, namely 4-hydroxynonenal (4-HNE) protein adducts derived from PUFA peroxidation. These authors investigated the modulation of the aortic proteome in atherosclerotic prone (LDLR^−/−^) mice fed an atherogenic diet supplemented with DHA for 20 weeks. Nineteen proteins were differentially regulated in the aorta of DHA-supplemented group. Most of them were related to glucose or lipid metabolism, including the up-regulation of superoxide dismutase by DHA which suggested an impact on vascular antioxidant defenses. This up-regulation was in agreement with data from the quantification of proteins with 4-HNE adducts. None of the twelve different identified proteins with 4-HNE adducts enhanced their oxidation in response to DHA supplementation. The articles cited in this section of the review are summarized in Table 1.

### 2.2. Beneficial Effects of Marine Lipids Intake Assayed by Lipidomics

In the era of genomics, transcriptomics and proteomics, metabolomics is becoming a critical component of the omics revolution and systems biology [9]. Among the four types of biological molecules that compose the human body, i.e., nucleic acids, amino acids, carbohydrates and lipids, the study of lipid homeostasis has been attracting increasing interest, especially during the last decade. As a subfield of metabolomics, lipidomics addresses the large-scale study of lipids, including the detailed characterization of lipid metabolites, their interactions and influence on biological systems. Lipidomics comprises the methodologies used for the study of a lipidome, which can be defined as the comprehensive and quantitative description of a set of lipid species present in an organism [29]. 

In spite of a lack of a commonly accepted definition, lipids are hydrophobic or amphipathic compounds of relatively small molecular weight. They include a vast plethora of different structures which play a critical role in cell physiology. Based on their chemical structure, lipids are divided into eight main classes: (a) fatty acyls, such as polyunsaturated fatty acids (PUFAs); (b) glycerolipids (GLs), such as triglycerides (TGs); (c) glycerophospholipids (GPs), also referred to as phospholipids (PLs), such as phosphatidylcholine; (d) sphingolipids (SPs), such as ceramides; (e) sterol lipids (STs) such as cholesterol; (f) prenol lipids (PRs); (g) saccharolipids (SLs); (h) polyketides (PKs). Some of the biological functions of these lipids are energy storage and structural components of cellular membranes, cell signaling, endocrine actions and essential role in signal transduction, membrane trafficking and morphogenesis [30]. 

Lipids are potent signaling molecules which can act as biosynthetic precursors of lipid mediators. In this review, the lipid mediators derived from ω-6 and ω-3 PUFAs, such as ARA, EPA, and DHA, are particularly noteworthy. These lipid mediators, which are generally known as eicosanoids (derivatives from the oxidation of C20 PUFAs, i.e., ARA, EPA, and dihomo-γ-linolenic acid (DGLA)) and docosanoids (derivatives from the oxidation of C22 PUFAs, i.e., DHA and docosapentaenoic acid (DPA)), are bioactive compounds related to inflammatory processes [31]. While most of them are formed by the action of lipoxygenases (LOX), cyclooxygenases (COX), cytochrome P450 (CyP450), and several subsequent enzymes on PUFAs, several compounds, for instance, isoprostanes, are the consequence of non-enzymatic process [32]. The main lipid mediators originated from ARA, EPA, and DHA are shown in Table 2. 

The enormous complexity of lipidomes implies that lipidomics must consider the characterization of thousands of pathways and networks which involve cellular lipids species and their interactions with other molecules. Lipidomics is a valuable tool to investigate the influence of marine lipids on health. However, its use is still limited due to its novelty but also to several difficulties associated with data interpretation, among other limitations [33]. In consequence, only a few studies have addressed the effect of marine lipids on the lipidome in the context of human metabolic disorders. Some of them used lipidomics to characterize total lipid classes, mainly by using gas chromatography (GC)-MS or LC-MS, and others were focused on lipid mediators, mainly by using solid phase extraction (SPE) coupled to identification and quantification by LC-MS/MS. 

By using Scopus database, 53 publications were found following these search criteria: TITLE-ABS-KEY (“omega 3” OR “PUFA” OR “eicosapentaenoic” OR “docosahexaenoic” OR “marine fatty acids” OR “marine lipid” OR “marine oil” OR “fish oil” OR “fish lipid” ) AND TITLE-ABS-KEY (nutrition OR diet) AND TITLE-ABS-KEY (“metabolic disorder” OR “metabolic alteration” OR “metabolic disease” OR obesity OR “metabolic syndrome” OR diabetes OR “cardiovascular disease” OR cholesterol OR insulin OR atherosclerosis OR inflammation OR “oxidative stress”) AND TITLE-ABS-KEY (lipidomic OR lipidomics OR lipidome). These publications, mainly research articles (69%) and reviews (19%), belong, in descending order of abundance, to the fields of medicine (65%) and biochemistry, genetics and molecular biology (61%), and to a lesser extent nursing, agricultural and biological sciences, pharmacology, toxicology and pharmaceutics, chemistry, immunology and microbiology, neuroscience, etc. 

In this section, the same inclusion criteria exposed above (Section 2.1) for proteomics articles is followed (i.e., only research articles focusing on lipidomics, which have assayed the effect of marine lipids on features of MetS, cardiovascular diseases and type 2 diabetes, excluding the studies which have used ω-3 PUFAs from vegetal origin, i.e., ALA). Likewise, works which have combined both proteomics and lipidomics approaches will be presented in Section 2.3. 

According to these filters, 35 research articles have been found and are summarized below.

#### 2.2.1. Lipidomics in Clinical Trials

Several researches have used lipidomics tools in human clinical trials to gain insights of physiological mechanisms behind the evidence of the multiple beneficial health effects of fish consumption.

Some of them have investigated the modulation of plasma/serum lipidome by fish oil in healthy subjects. Ottestad et al. [34] studied the plasma lipidomic profile in healthy subjects which received either 8 g/day of fish oil from cod liver or high oleic sunflower oil for 7 weeks. Authors identified and quantified 260 different lipids in plasma, being 23 lipids significantly decreased and 51 significantly increased by fish oils. Data analysis demonstrated that fish oil supplementation altered lipid metabolism and increased the plasma proportion of PLs and TGs containing long-chain PUFAs. Therefore, the beneficial effects of fish oil supplementation could be explained in part by a remodeling of the plasma lipids. Rudkowska et al. [35] combined transcriptomics and metabolomics technologies to investigate molecular and metabolic changes in healthy subjects underwent 6-week supplementation with EPA and DHA. Authors measured 107 lipids in plasma, which were further subdivided into three different classes: 15 sphingomyelins (SMs) and SM derivatives, 15 lysophosphatidylcholines (lysoPCs) and 77 glycerophosphatidylcholines (glyPCs). Results showed some gender differences in lipidomic profiles between pre- and post- ω-3 supplementation, although the main differences were found as a result of fish oil supplementation. These differences were principally due to changes in glyPCs, and overall, results demonstrated that there was an increase in unsaturated fatty acids after the ω-3 PUFAs supplementation period. These and others data given by authors supported the cardioprotective effects of the ω-3 PUFAs supplementation, although some of their mechanisms can be dependent on gender. The high variability in lipid profiles and lipidome responses to PUFAs supplementation was addressed in a third study performed by Nording et al. in healthy subjects. These authors used a multi-platform lipidomics approach to investigate both the consistent and inconsistent responses to a defined ω-3 intervention for 6 weeks [36]. Thus, Nording et al. evaluated the changes induced by ω-3 intervention in total lipidomic plasma profile (including fatty acids, lipid classes and lipoprotein distribution) but also the effects of ω-3 on lipid mediators. Authors measured 7 lipid classes, and a total of 87 lipid mediators. Results showed significant changes in both total lipidomic and lipid mediator profiles after ω-3 supplementation, as well as a strong correlation between lipid mediator profiles and EPA and DHA incorporated into different lipid classes. However, authors found that both ω-3 and ω-6 fatty acid metabolites displayed a large degree of variation among the subjects; for instance, only the 50% of the subjects presented significantly decreased levels of PGE_2_, TXB_2_ and 12-HETE whereas the other 50% did not show any change or even increased levels. Specifically, 12-HEPE showed high heterogeneity, decreasing up to 82% in some subjects and increasing up to 5% in others. This work pointed out the highly variable response to ω-3 fatty acids supplementation and the need for an in-depth lipidomic phenotype characterization in order to properly assess their effectiveness against diseases. 

Since lipidomic characterization requires an accurate determination of the huge range of lipid mediators and fatty acid derivatives, some authors have tried to shed light on this matter. Mas et al. [37] published the development of a SPE-LC-MS/MS assay to measure resolvins and protectins families generated from the ω-3 EPA and DHA in human blood after fish oil supplementation (4 g fish oil containing 35% EPA and 25% DHA/day for 3 weeks). It was the first time that 17R/SHDHA RvD_1_ and RvD_2_ were detected in plasma/serum after oral ω-3 fatty acid supplementation. Authors found that those RvD_1_ and RvD_2_ were within the biological range of anti-inflammatory and pro-resolving activities detected in isolated human leukocytes and in vivo studies in mice. This methodology was further employed to examine the effect of short-term (5 days) ω-3 fatty acid supplementation. As compared to baseline, ω-3 intake significantly increased plasma levels of RvE_1_, 18R/S-HEPE, 17R/S-HDHA, and 14R/S-HDHA up to concentrations biologically active in healthy humans. Therefore, ω-3 PUFAs were able to exhibit their anti-inflammatory action even after short interventions [38]. 

Furthermore, Keelan et al. [39] determined if the supplementation with EPA and DHA during pregnancy could modify placental PUFAs composition and the accumulation of lipid mediators. In this case, only resolvins (RvD_1_, 17R-RvD_1_ and RvD_2_) and protectins from the D-series (PD_1_ and 10S, 17SdiHDHA) and upstream precursors (18-HEPE and 17-HDHA) were measured. Authors found that the ω-3 PUFAs supplementation increased placental DHA levels, as well as the levels of precursors 18-HEPE and 17-HDHA, but the concentration of EPA was not significantly increased neither concentrations of RvD_1_, 17R-RvD_1_, RvD_2_ and PD_1_. Placental pro-resolving lipid mediator levels seemed to be modulated by maternal dietary PUFAs, although their biological significance in the placenta remains unknown. 

Besides healthy subjects, other authors have used lipidomics tools to evaluate changes in lipid homeostasis induced by ω-3 in patients suffering metabolic disorders. The modulation of lipid mediators by EPA and DHA was analyzed in plasma of humans with MetS [40]. Dietary intervention consisted in a daily supplementation with EPA and DHA in the form of TGs for 3 weeks. Plasma lipid mediators (i.e., 18-HEPE, E-series resolvins, 17-HDHA, D-series resolvins, 14-HDHA, and maresin-1) from MetS volunteers and their healthy controls (at baseline and after dietary intervention) were measured. Results showed that ω-3 PUFAs supplementation increased E-series resolvins to a similar extent in MetS subjects and controls. However, only the healthy controls presented increased concentrations of E- and D-series resolvin precursors and 14-HDHA in response to ω-3 PUFAs supplementation. The action of EPA and DHA supplementation was also investigated in hyperlipidemic men (cholesterol >200 mg/dL; triglyceride >150 mg/mL) after 12-weeks daily intake [41]. Schuchardt et al. measured serum levels of 44 free hydroxy, epoxy and dihydroxy fatty acids and found that after supplementation, all subjects (including healthy controls) showed considerably elevated levels of EPA-derived lipid mediators and a less pronounced increment of DHA-derived ones. However, the supplementation with higher amounts of DHA than EPA (DHA:EPA 5:1) for 3 months induced a significant increase of pro-resolving DHA derivatives in plasma of obese women [42]. All these different results in the level of EPA and DHA derivatives seemed to be correlated with the EPA and DHA supplement content. 

In another study, Lankinen et al. [43] investigated how fatty fish or lean fish in a diet affect serum lipidomic profiles in subjects with coronary heart disease after their consumption for 8 weeks. Lipidomic changes among groups were detected in at less 59 bioactive lipid plasma species, including ceramides, lysoPCs and diacylglycerols (DGs), which were found significantly diminished in the fatty fish group, whereas in the lean fish group cholesterol esters and specific long-chain TGs increased significantly. Therefore, fatty fish intake reduced lipid species which are potential mediators of lipid-induced insulin resistance and inflammation, and these results might be associated with the protective effects of fatty fish on the progression of atherosclerotic vascular diseases or insulin resistance. However, Midtbø et al. [44] demonstrated in mice fed western diets that the consumption of farmed salmon, which had previously fed fish feed with a reduced ratio of ω-3/ω-6 PUFAs, led to a selectively increased abundance of ARA in the liver PLs pool of the mice. This increment was accompanied by higher levels of hepatic ceramides and ARA-derived pro-inflammatory mediators and a reduced abundance of lipid mediators derived from EPA and DHA. Therefore, the studies made after fish consumption rather than fish oil have to consider the PUFA composition of fish to get proper conclusions. 

Two articles have used enriched ω-3 PUFAs dairy products to test their effects on lipidome. In the first one, mildly hypertriacylglycerolemic subjects consumed yogurt supplemented with 3 g of EPA and DHA per day for 10 weeks [45]. Results showed that a daily intake of supplemented yogurt significantly increased plasma EPA-derived mediators (PGE_3_, 12-, 15-, 18-HEPE), as well as EPA and DHA levels in plasma and red blood cells, and improved some cardiovascular risk factors. In the second one, overweight and moderately hypercholesterolemic subjects consumed 250 mL of enriched milk with EPA and DHA for 28 days [46]. In this case, the changes induced on LDL-lipidome composition were primarily addressed. Enriched milk significantly reduced TGs and very low-density lipoprotein (VLDL) cholesterol and caused significant changes in the LDL lipid metabolite pattern, increasing the long-chain polyunsaturated cholesteryl esters and the ratio PC36:5/lysoPC16:0. All these modifications were associated with its reduced inflammatory activity.

#### 2.2.2. Lipidomics in Animal Models

In animal models, several authors have investigated the influence of dietary EPA and DHA on lipid homeostasis in the context of metabolic alterations through lipidomics approaches. Some of them have used healthy models to look into the potentially different effects of EPA and DHA to find their optimal proportions in the diet. Dasilva et al. [47] tested whether the intake of three proportions of EPA/DHA (1:1, 2:1 or 1:2) for 22 weeks provoked a different modulation of the formation of lipid mediators. Then, authors examined their influence on various indexes of inflammation and oxidative stress in Wistar Kyoto rats. A total of nine compounds derived from PUFA oxidative metabolism (namely five EPA eicosanoids -12HEPE, 15HEPE, 12HpHEPE, 15HpHEPE, TXB_3_-, two DHA docosanoids -17HDoHE, 17HpDoHE-, and two ARA eicosanoids -11HETE, PGE_2_) were identified and quantified in plasma. Results evidenced that the ratios 1:1 and 2:1 EPA:DHA exerted a remarkable healthy effect generating a less oxidative environment and modulating LOX and COX activities towards a decrease in the production of pro-inflammatory ARA eicosanoids and oxidative stress biomarkers from EPA and DHA. On the other hand, the higher DHA amount in the diet (i.e., 1:2 ratio) reduced the health benefits described in terms of inflammation and oxidative stress. The beneficial effect of the ratios with higher EPA amount was further evaluated in a rat model of MetS (SHROB rats), in which the 1:1 and 2:1 ratios exerted the highest health benefits. In this other work, EPA and DHA supplementation also decreased the level of pro-inflammatory ARA eicosanoids produced, in agreement with the results found in the healthy model [48]. Other authors [49] reported that the benefit of ω-3 PUFA-rich diets could be attributed to the generation of electrophilic oxygenated metabolites that transduce anti-inflammatory actions rather than the suppression of pro-inflammatory ARA metabolites. This work was focused on the endogenous production of ω-3 PUFAs electrophilic ketone derivatives and their hydroxy precursors in human neutrophils. Authors evaluated in vitro endogenous generation of these lipid mediators from DHA and DPA in neutrophils isolated from healthy subjects, both at baseline and upon stimulation with calcium ionophore. Additionally, their potential modulation by diet was assessed through a randomized clinical trial carried out with healthy adults receiving daily oil capsule supplements, which contained either 1.4 g of EPA and DHA or soybean oil, for 4 months. Results reported the 5-LOX-dependent endogenous generation of 7-oxo-DHA, 7-oxo-DPA and 5-oxo-EPA and their hydroxy precursors stimulated in human neutrophils, whereas the dietary supplementation with EPA and DHA increased the formation of 7-oxo-DHA and 5-oxo-EPA, without significant modulation of ARA metabolite levels. 

In C57BL/6 mice fed a diet containing 10% ω-3 PUFAs from menhaden oil for 4 months, Balogun et al. [50] analyzed the effect of fish oil on the fatty acid composition of various bioactive lipids in plasma and liver by using lipidomics. Results demonstrated a significantly higher concentration of EPA containing phosphatidylcholine (PCs), lysophosphatidylcholine (LPCs), and cholesteryl esters (CEs) after fish oil intake in plasma and liver, as well as a higher concentration of free ω-3 PUFAs. 

In healthy rats, lipidomics was also used to determine if LOX-generated lipid mediators were presented in bone marrow and if so, their modulation by dietary EPA and DHA supplementation [51]. Data analysis revealed the presence of LOX-pathway lipid mediators derived from ARA, EPA and DHA, including lipoxins, resolving D_1_, resolvin E_1_, and protectin D_1_ in bone marrow. Moreover, the daily supplementation with DHA or with EPA ethyl ester for 4 months increased the percentage of DHA and EPA in bone marrow, and the proportion of LOX mediators biosynthesized from DHA or EPA, respectively. Given the potent bioactivities of the lipoxins, resolvins and protectins, their presence and changes in their profile found after EPA and DHA ethyl ester supplementation may be of interest in bone marrow function and as a potential source of these mediators in vivo.

Several articles have used lipidomics to assay the protective effects of EPA and DHA against metabolic alterations induced by unhealthy diets. In rats, Taltavull et al. [52] investigated how supplementation of a high fat and sucrose diet with both EPA and DHA modified the hepatic ceramide profile triggered by the unhealthy diet in a dietary intervention of 24 weeks. Authors found that ω-3 PUFAs reduced total liver ceramide content and altered ceramide profiles in pre-diabetic rats. They also observed a significant positive linear correlation between long chain ceramide 18:1/18:0 and the HOMA index, and negative between very long chain ceramides 18:1/24:0 and 18:1/20:0 and plasma insulin levels and the HOMA index. Overall, these data may help explain the protective action of ω-3 PUFAs against liver insulin resistance induced by diet. Caesar et al. [53] used lipidomics tools to evaluate the regulation of lipid composition in mice liver and serum by dietary fish oil as compared to lard oil, but considering their interaction with gut microbiota. After 11 weeks, menhaden fish oil supplementation induced significant changes in abundance of most lipid classes. The gut microbiota affected lipid composition by increasing hepatic levels of cholesterol and cholesteryl esters in mice fed high-fat lard diet but not in mice fed high-fat fish oil diet. These results highlighted that the regulation of hepatic cholesterol metabolism induced by gut microbiota was dependent on dietary lipid composition. 

Animal models of obesity have also been used to investigate the modulation of lipid metabolism in the adipose tissue. Kuda et al. [54] identified cells producing lipid mediators in epididymal WAT of mice fed for 5 weeks obesogenic high-fat diet, which was supplemented or not with EPA and DHA. Results demonstrated selectively increased levels of anti-inflammatory lipid mediators in WAT in response to ω-3, reflecting either their association with adipocytes (endocannabinoid-related Ndocosahexaenoylethanolamine) or with stromal vascular cells (pro-resolving lipid mediator protectin D_1_). In parallel, tissue levels of obesity-associated pro-inflammatory endocannabinoids were suppressed. Moreover, they found that adipose tissue macrophages (ATMs) were not the main producers of protectin D_1_ and that ω-3 PUFAs lowered lipid load in ATMs while promoting their less-inflammatory phenotype. Besides these specific roles of various cell types in WAT, the kind of fat depots seemed to be also critical. In the abdominal (epididymal) fat but not in other fat depots, Flachs et al. [55] found a synergistic induction of the mitochondrial oxidative capacity and lipid catabolism after combining ω-3 PUFAs intake and caloric restriction in high-fat fed mice. This combination resulted in an increased oxidation of metabolic fuels in the absence of mitochondrial uncoupling, while low-grade inflammation was suppressed, reflecting changes in tissue levels of anti-inflammatory lipid mediators, namely 15-deoxy-Δ(12,15)-prostaglandin J_2_ and protectin D_1_.

Lipidomics analysis [56] also revealed that ω-3 PUFAs supplementation could alleviate hepatic steatosis in *ob/ob* mice, an obesity model of insulin resistance and fatty liver disease. ω-3 PUFAs inhibited the formation of ω-6 PUFAs derived eicosanoids while triggering the formation of ω-3 PUFAs derived resolvins and protectins. Moreover, representative members of these lipid mediators, namely resolvin E_1_ and protectin D_1_, mimicked the insulin sensitizing and anti-steatotic effects of ω-3 PUFAs and induced adiponectin expression to a similar extent that the antidiabetic drug rosiglitazone. These findings uncovered beneficial actions of ω-3 PUFAs and their bioactive lipid derivatives in preventing obesity-induced insulin resistance and hepatic steatosis. Similar conclusions were obtained by Kalish et al. [57] who demonstrated in a mouse model of steatosis that parental nutrition with fish oil-based lipid emulsions was associated with the production of anti-inflammatory and pro-resolving lipid mediators. The preventive effect of EPA and DHA against necroinflammatory injury in liver was also investigated in mice fed high saturated fat diets containing either DHA or both EPA and DHA for 5 weeks [58]. Both marine ω-3-rich diets induced an increased hepatic formation of DHA-derived lipid mediators (i.e., 17*S*-hydroxy-DHA (17*S*-HDHA) and protectin D_1_), which was correlated with significant protection of liver injury. This work reported a potential role for DHA-derived products, specifically 17*S*HDHA and protectin D_1_, in mediating the protective effects of dietary DHA against necroinflammatory liver injury.

The potential role of fish oil to prevent glomerulosclerosis in a rat model of MetS (JCR:LA-cp rats) via renal eicosanoid metabolism and lipidomics analysis was addressed by Aukema et al. [59]. MetS rats were supplemented with 5% or 10% fish oil for 16 weeks. Dietary fish oil reduced glomerulosclerosis and albuminuria and the 11- and 12-HETE levels, as well as other (5-, 9- and 15-) HETE. Also, fish oil reduced endogenous renal levels of 6-keto PGF_1α_ (PGI_2_ metabolite), thromboxane B_2_ (TXB_2_), PGF_2α_, and PGD_2_ by approximately 60% in rats fed 10% fish oil as compared to untreated MetS rats. Whereas in rats fed 5% fish oil, TXB_2_ decreased in 250% and PGF2a in 241%. These results suggested that dietary fish oil might improve dysfunctional renal eicosanoid metabolism associated with kidney damage during conditions of the MetS. 

The impact of DHA supplementation on the profiles of PUFA oxygenated metabolites and their contribution to atherosclerosis prevention were investigated by Gladine et al. [60]. The study was conducted with atherosclerosis prone mice which received increasing doses of DHA (0%, 0.1%, 1% or 2% of energy) during 20 weeks. Targeted lipidomics analysis determined a significant modulation of EPA and DHA and their respective oxygenated metabolites in plasma and liver. Remarkably, hepatic F4-neuroprostanes were strongly correlated with the hepatic DHA level. The hepatic level of F4-neuroprostanes was the variable most negatively correlated with the plaque extent and plasma EPA-derived diols. Thus, oxygenated ω-3 PUFAs derivatives, particularly F4-neuroprostanes, were revealed as potential biomarkers of DHA-associated_atherosclerosis prevention which might contribute to the anti-atherogenic effects of DHA.

It is well known that oils from marine organisms have a different fatty acid composition and differ in their molecular composition. Fish oil has a high content of EPA and DHA mostly esterified to TGs, while in krill oil these fatty acids are mainly esterified to PLs. Considering that, Skorve et al. [61] studied the effects of these oils on the lipid content and fatty acid distribution in the various lipid classes in liver and brain of mice. After 6 weeks of feeding a high-fat diet supplemented with fish oil or with krill oil, shotgun lipidomics showed that in both fish and krill oil fed mice, the TGs content in the liver was more than doubled compared to control mice. The fatty acid distribution was affected by the oils in both liver and brain with a decrease in the abundance of LA and ARA, and an increase in EPA and DHA in both study groups. LA decreased in all lipid classes in the fish oil group but with only minor changes in the krill oil one. Differences were especially evident in some of the minor lipid classes associated with inflammation and insulin resistance. Ceramides and DGs were decreased, and cholesteryl esters increased in the liver of the krill oil group, while plasmalogens were diminished in the fish oil group. In the brain, DGs were decreased, more by krill than fish oil, while ceramides and lactosylceramides were increased, more by fish than krill oil. Changes in hepatic sphingolipids and ARA fatty acid levels were higher in the krill oil group than in the fish oil one. These changes were consistent with a hypothesis that krill oil may have a stronger anti-inflammatory action and enhance insulin sensitivity more potently than fish oil. 

#### 2.2.3. Lipidomics in Cell Cultures

Finally, some authors have applied lipidomics approaches in cell cultures and in vitro assays to deeply analyze the metabolic effect of dietary marine ω-3 PUFAs. Polus et al. demonstrated that the addition of EPA during differentiation of human subcutaneous adipose tissue stromal vascular fraction cells induced the formation of small lipid droplets and reduced the production of pro-inflammatory mediators in adipose tissue in comparison to ARA addition. These changes were the consequence of the production of anti-inflammatory eicosanoids derived from EPA [62].

The preventive effect of DHA at physiological doses against insulin resistance was investigated in C2C12 myotubes exposed to palmitate. DHA decreased protein kinase C activation, restored cellular acylcarnitine profile, insulin-dependent AKT phosphorylation and glucose uptake. Results showed that DHA participated in the regulation of muscle lipid and glucose metabolism by preventing lipotoxicity, inflammation and insulin resistance in skeletal muscle [63]. Likewise, in in vitro experiments, Ting et al. [64] investigated structural changes in cardiolipins after DHA or EPA supplementation and compared them to ARA treatment, using H9c2 cardiac myoblast as a cell model. Among the 116 cardiolipin species with 36 distinct mass identified, the three PUFAs treatments differentially perturbed the fatty acyl chain compositions in the mitochondrial of the H9c2 cardiac myoblast, suggesting that both mitochondrial membrane composition and function were susceptible to exogenous lipids. Additionally, DHA supplementation correlated with an elevation of less unsaturated and ω-3 cardiolipin species, which appeared to be a minor effect on EPA but not on ARA.

#### 2.2.4. Marine Lipids and Other Bioactive Compounds Assayed by Lipidomics

Several articles have also used lipidomics to study the combined action of ω-3 PUFAs with other bioactive compounds which have shown potential benefits in subjects susceptible to cardiovascular diseases. 

The protective effects of fatty fish consumption on the progression of insulin resistance were tested in combination with other products with recognized effect on glucose metabolism (whole grain and low postprandial insulin response grain products, and bilberries) in a clinical trial [65]. Plasma lipidomic profiles of people with impaired glucose metabolism and with at least two other features of the MetS were evaluated after 12 weeks of dietary intervention. Among the 364 characterized lipids in plasma, 25 changed significantly in the treated group, including multiple TGs incorporating the long chain ω-3 PUFAs. These results were supported by biochemical data and suggested an improvement of glucose metabolism and a beneficial effect in preventing type 2 diabetes in population groups at considerably higher risk of suffering it. 

Other three articles found in literature have employed lipidomics tools to study the combined action of ω-3 PUFAs with other bioactive compounds which have shown potential benefits in subjects suffering CVD or at considerably high risk. The first one [66] was designed to assay the combined effect of l-alanyl-l-glutamine and fish oil supplementation for 3 months on skeletal muscle function and metabolism in patients with chronic heart failure. Patients were randomized to either l-alanyl-l-glutamine and PUFAs or placebo (safflower oil and milk powder). Regular uptake of the bioactive compounds led to the expected increase in unsaturated fatty acids. Moreover, the lipidomic analysis revealed a decrease in circulating levels in total ceramides and two ceramide subspecies (C22:1 and C20:1) induced by supplements at 4 weeks, which was not detectable in samples at 3 months. In another study, the combined effect of ω-3 fatty acids with Coenzyme Q10 was evaluated on plasma lipid mediators profile in patients with chronic kidney disease (CKD), which are highly predisposed to suffer CVD, partially due to their chronic inflammation [67]. Patients received a daily dose of ω-3 PUFAs, Coenzyme Q10 (CoQ), or both supplements for 8 weeks. Compounds as 18-HEPE, 17-HDHA, RvD_1_, 17R-RvD_1_, and RvD_2_ were measured in plasma before and after the intervention. Results showed that ω-3 PUFAs but not CoQ significantly increased plasma levels of the upstream precursors of the E and D-series resolvins (18-HEPE and 17-HDHA, respectively) as well as RvD_1_. This finding may have important implications for limiting ongoing low-grade inflammation in CKD. Finally, Bondía-Pons et al. [68] investigated the effects of ω-3 PUFAs and polyphenol rich diets on plasma and HDL fraction lipidomic profiles in MetS patients. Authors compared the effects of diets contained low or high ω-3 PUFAs (EPA and DHA) in combination with low or high of polyphenols, resulting in 4 isoenergetic diets, differing in their natural ω-3 PUFAs and polyphenols amount. Authors successfully identified 350 and 293 lipid species in total plasma and HDL fraction samples respectively. Results showed that the two diets high in ω-3 PUFAs highly increased unsaturated long-chain TGs and EPA and DHA-containing PLs levels and decreased levels in total plasma of low unsaturated PLs, and PCes, LysoPCs, and PCps with ARA in their structure. With regards to HDL, PCs and TGs with DHA or EPA in their structure increased after the consumption of high ω-3 PUFAs diets, while PCes and PCps with ARA in their structure, and medium-chain PCs decreased. The diet high in both ω-3 and polyphenols significantly reduced PCs and PEs levels, especially of those alkyl and alkenyl ether lipids with 16:0 in their structure as well as saturated and low-unsaturated PCs and PEs. The study found a relevant association among lipidomics data, dietary and clinical/anthropometric variables. The most remarkable and complex association among variables was observed after the intervention with the diet high in both ω-3 PUFAs and polyphenols. Different types of TGs were positively or negatively associated with waist circumference, which was positively associated with insulin levels. Glucose was positively associated with body weight, which was negatively associated with EPA. This latter association was only detected after feeding the combined diet, which may mean that dietary polyphenols interacted with ω-3 PUFAs in the regulation of body weight in MetS subjects. Overall, data reflected different lipid rearrangements after a nutritional intervention with diets rich in ω-3 PUFAs and polyphenols in these patients at a high CVD risk.

The promising cooperative effect between fish oil and polyphenols was further analyzed considering the ability of polyphenols as antioxidants to potentially ameliorate oxidative damage of ω-3 PUFAs when they are consumed together and to enhance their individual potential effects on metabolic health through the modulation of fatty acids profiling and the formation of lipid mediators. Dasilva et al. [69] evaluated the effect of diet supplementation with EPA and DHA, grape polyphenols or both in rats fed either standard or high fat and sucrose diets (a total of eight diets), on the inflammatory response and redox unbalance triggered by these unhealthy diets. Authors analyzed total fatty acid composition in the liver, plasma, adipose tissue, erythrocytes as well as circulating FFA in plasma across experimental groups and calculated fatty acid desaturases (FADs) indexes (stearoyl-CoA desaturases SCD-16 and SCD-18 as well as desaturases Δ4, Δ5, and Δ6). Likewise, a total of nine compounds derived from PUFA oxidative metabolism (namely five EPA eicosanoids, two DHA docosanoids and two ARA eicosanoids) were identified and quantified in plasma. Data analysis reflected that the supplementation with fish oil led to an anti-inflammatory situation associated with a lower ω-6/ω-3 index in plasma and membranes, a lower production of ARA pro-inflammatory lipid mediators, an up-regulation of desaturases related to EPA and DHA synthesis and a down-regulation of these desaturases to synthesize ARA. However, polyphenols bioactivity was influenced by the background diet. In a standard diet, they seemed to modulate enzymes towards an anti-inflammatory and antioxidant response, and the combination with fish oil down-regulated Δ5D related with ARA synthesis, decreased COX activity on ARA, enhanced the antioxidant enzymes and decreased total FFA in plasma. Similarly, the combination of both supplements also produced a significant improvement in the antioxidant balance and oxidative stress in unhealthy diets. However, the efficacy of polyphenols to reduce inflammation was lower when were added to the unhealthy diet, and some pro-inflammatory pathways were found even up-regulated. Therefore, fish oil seemed to be the main responsible for the anti-inflammatory effects observed in the combined group in the unhealthy diet. The combination of both bioactive supplements may improve the metabolic health in both background diets by acting on inflammation and oxidative stress pathways. A summary of the articles cited in this section of the review is shown in Table 3.

### 2.3. Beneficial Effects of Marine Lipids Intake Assayed by Both Proteomics and Lipidomics

The number of research articles which have combined both proteomics and lipidomics approaches to address the beneficial effects of marine lipids in metabolic alterations induced by unhealthy diet intake is scant yet. More articles have used genomics or transcriptomics tools, but gene expression or transcription is not always correlated with protein levels or activities. The application of proteomics and lipidomics approaches can help overcome this drawback and identify molecular pathways actually affected by marine lipids. The integration of different omics seems essential to have a complete picture of how marine lipids affect health. In spite of this observation, only three research articles which have combined both proteomics and lipidomics are currently available.

The first study was carried out in healthy overweight men with mildly elevated plasma C-reactive protein concentrations. Bakker et al. [70] performed a dietary intervention with a mix of several products selected for their evidence-based anti-inflammatory properties. That “anti-inflammatory dietary mix” consisted of fish oil, green tea extract, resveratrol, vitamin E, vitamin C, and tomato extract. Regarding fish oil, subjects consumed 1200 mg cold water fish oil/day, composed of 380 mg EPA and 260 mg DHA, and 60 mg other ω-3 PUFAs. Authors measured inflammatory and oxidative stress defense markers in plasma and urine. Furthermore, 120 plasma proteins, 274 plasma metabolites (lipids, FFA, and polar compounds) and the transcriptomes of peripheral blood mononuclear cells and adipose tissue were also quantified. Therefore, this study combined proteomics, metabolipidomics and transcriptomics tools. Plasma adiponectin concentrations increased by 7%, whereas C-reactive protein (principal inflammation marker) was unchanged. However, a multitude of subtle changes was detected by an integrated analysis of the omics data, which indicated modulated inflammation of adipose tissue, improved endothelial function and oxidative stress, and increased liver fatty acid oxidation. Using the same subjects, Pellis et al. [71] determined their postprandial response to the consumption of a standardized 500 mL high-fat dairy shake by using metabolomics and proteomics tools. During a 6 h time course after Postprandial Challenge Test (PCT), authors quantified several plasma metabolites, 79 plasma proteins and 7 clinical biochemistry parameters (glucose, insulin, total FFA, total TGs, hsCRP, IL6, and TNFa). Among these, 31 had different responses over time between treated and control groups, revealing differences in amino acid metabolism, oxidative stress, inflammation, and endocrine metabolism. Results showed different short-term metabolic responses to the PCT in subjects previously supplemented with the anti-inflammatory mix compared to the controls. Additional metabolic changes related to the dietary intervention were also detected as compared to non-perturbed conditions. 

In the third research [72] the obesogenic effect of diets heavily enriched in ω-6 PUFAs and poor in ω-3 PUFAs was assayed by combined proteomics and lipidomics. The study was carried out in aging mice, which had previously suffered a myocardial infarction, after 5 months of feeding a ω-6 enriched diet (ω-6:ω-3 442:1 ratio). Plasma proteomic profiling revealed higher VCAM-1, macrophage inflammatory protein-1 and D40 and myeloperoxidase in the ω-6 PUFAs group. Lipidomic analysis showed higher levels of ARA and 12(S)-HETE and altered levels of inflammation-resolving enzymes 5-LOX, COX-2, and heme oxygenase-1, which reflected that excess of ω-6 stimulate prolonged neutrophil trafficking and pro-inflammatory lipid mediators after myocardial infarction. Table 4 summarizes the articles cited in this section of the review.

## 3. Mechanisms behind the Beneficial Effects of Marine Lipids Assayed by Proteomics and Lipidomics

The information obtained from both proteomics and lipidomics approaches has confirmed mechanisms proposed by genomics and transcriptomics data on the role of marine lipids in diet-induced metabolic diseases. But interestingly, these techniques have also provided new insights suggesting the modulation of new molecular pathways and proteins.

Lipidomics tools have revealed the potential mechanisms related to the influence exerted on the regulation of lipid profiles in plasma/blood, tissues and membranes and lipid mediator synthesis by marine lipids in human, animal and cell-model experiments. These lipid profiles and their derivative metabolites from fatty acids have closely been associated with inflammation, oxidative stress and the endogenous antioxidant system [73,74].

Besides their known effects in decreasing plasma TGs and cholesterol levels, some of the lipidomics studies reported in this review have found a remodeling of the plasma/membrane/tissue lipids into PLs, TGs, lipoproteins and other lipid species of long chain PUFAs, including plasma FFA profiles [34,35,36,39,43,44,45,46,47,48,50,51,52,53,60,61,63,64,65,66,68,69]. In general, the consumption of marine ω-3 led to a replacement of ARA with EPA and DHA in cell membranes and lipid species presented in plasma, erythrocytes and liver and adipose tissue, but also kidney and muscle. Such replacement provoked the consequent enrichment on ω-3 long chain PUFAs accompanied by modulation of LOX and COX activities, which was attributed to a competence mechanism, especially between ARA and EPA. As a result, the production of lipid mediators was affected. Additionally, the uptake of ω-3 PUFAs modulated the synthesis de novo of ARA through elongases and desaturases. In fact, lipidomics data from the liver, which is in charge of the de novo fatty acids synthesis, confirmed the preferential substrate competition of Δ5D, which controls de novo synthesis of EPA and ARA, for ω-3 PUFAs over ω-6 PUFAs [44,50,53,60,61,69]. Marine lipids also regulated the formation of bioactive lipids such as ceramides. The level of long chain ceramides associated with insulin resistance was found to be reduced in plasma [43,66] and liver [52,61] together to an increment of the concentration of very long chain ceramides which seemed to be IR protective.

The fatty acids modulation in the liver due to the consumption of marine lipids was reflected in the total fatty acids profile of plasma and adipose tissue [34,35,36,43,45,46,47,48,50,53,65,68,69]. Such modulation was also observed in circulating plasma FFA and the incorporation of fatty acids into erythrocyte membranes [47,48,69]. Fatty acids released from the adipose tissue, which become circulating FFA in plasma revealed the influence of the diet and synthesis de novo in the accumulation of fat in adipocytes. In consequence, the ω6/ω3 ratio in plasma, circulating FFA, membranes and tissues was lower after feeding marine lipids. The ω6/ω3 ratio is an excellent clinical marker for cellular inflammation [75] and higher values are correlated with increased prevalence of chronic inflammatory diseases [76]. 

In agreement with these effects, lipidomics studies focused on the formation of lipid mediators from marine PUFAs demonstrated that the intake of these lipids promoted the generation of anti-inflammatory and pro-resolving lipid mediators derived from EPA and DHA while decreasing pro-inflammatory mediators derived from ARA [37,38,39,40,41,42,44,45,47,48,49,51,54,55,56,57,58,60,62,64,67,69]. This anti-inflammatory response can be further due to the fact that ω-3 PUFAs, especially EPA, and ω-6 PUFAs can compete for the same enzymes, including phospholipases, desaturases, lipoxygenases and cyclooxygenases, resulting in a higher production of derived metabolites from EPA and DHA than derived from ARA. 

By using genomics and transcriptomics tools, some authors had previously reported that PUFAs inhibited the nuclear factor kb (NF-κB) and reduced cytokine production because EPA and DHA can inhibit the binding between saturated fats and toll-like receptors of membranes by direct competition. The binding to saturated fats would activate NF-κB gene transcription factor, which induces inflammatory responses through COX and cytokine synthesis [77]. Moreover, although it is not fully known, marine ω-3 PUFAs may also control gene expression by direct interaction with at least another 4 metabolic nuclear receptors: PPAR (peroxisome proliferator activated receptor), LXR (liver X receptor), HNF-4α (hepatic nuclear factor 4) and farnesol X receptor (FXR). Likewise, marine PUFAs can reduce the levels of sterol regulatory element binding proteins (SREBPs) and the carbohydrate response element binding protein (ChREBP) [78]. Therefore, the modulation of EPA and DHA levels and their outcomes revealed by lipidomics approaches may explain their actions in regulating gene expression. In addition to these findings, the use of proteomics approaches has confirmed the influence of EPA and DHA on several pathways modulated by these transcriptional factors. Interestingly, proteomics data identified specific proteins with a pivotal role in these pathways which were altered by marine lipids. In this regard, it is necessary to highlight that a direct correlation between the level of gene expression and the cellular content of proteins cannot always be found [79]. Consequently, proteomics becomes a critical tool for understanding marine lipid actions on cellular metabolism and for identifying biomarkers of modulation. Proteomics has specifically revealed that the influence of marine lipids intake is not limited to regulating cellular protein quantity but also protein quality by controlling the formation of oxidative PTMs on proteins, mainly carbonyl moieties. The control of these oxidative PTMs plays a key role in understanding the mechanism behind the beneficial effect of marine lipids in decreasing the risk of CVD and type 2 diabetes [80]. 

In the adipose tissue, aorta and especially liver, proteomics analysis revealed that ω-3 PUFAs from fish oil produced an improvement of lipid profiles and lower accumulation of fat through a mechanism that involved the down-regulation of proteins participating in lipogenesis and glycolysis while causing the up-regulation of proteins involved in fatty acid beta-oxidation. Fish oil also showed an important action on proteins implicated in the urea cycle and protein metabolism. Additionally, EPA and DHA demonstrated to act on insulin signaling by modulating proteins such as proteasome system [16,19,20,21,22,23,24,27,28]. 

Proteomics also found a substantial up-regulation of the antioxidant system in blood/plasma and the rest of tissue analyzed. In fact, fish oil induced higher levels of antioxidant enzymes, ameliorating of endoplasmic oxidative stress and stimulating protein and DNA cellular system repair [14,15,16,17,18,19,20,21,22,23,25,27,28]. Moreover, EPA and DHA altered protein carbonylation levels of specific liver proteins, demonstrating an additional mechanism of protein regulation by marine lipids, which is particularly interesting in the investigation of diseases induced by diet. It is important to point out that the impairment of normal redox homeostasis, and the consequent accumulation of oxidized biomolecules, has been linked to the onset and/or development of a great variety of diet-induced diseases [81,82]. Fish oil reduced carbonylation of proteins related to the antioxidant system such as albumin or 3-α-hydroxysteroid dehydrogenase in plasma and liver, respectively. Proteomics findings in liver demonstrated that EPA and DHA improved ammonia detoxification by decreasing carbonylation level of argininosuccinate synthetase while increasing oxidation of aspartate aminotransferase. Finally, in skeletal muscle, fish oil intake exerted a protection from cellular dysfunction by ameliorating actin carbonylation level [27]. 

**Figure 1:**
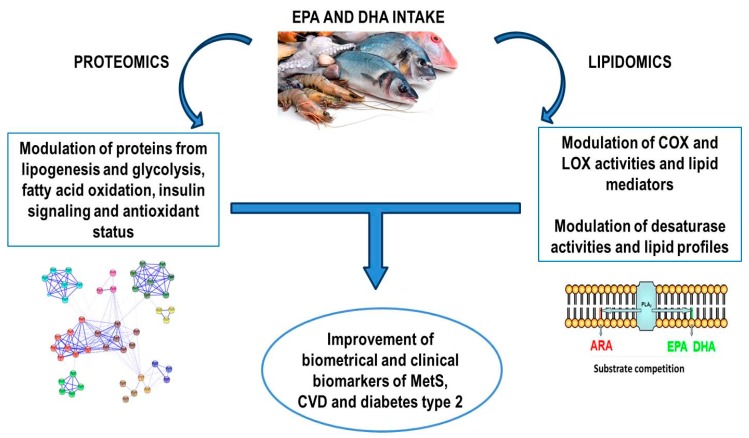
Schematic representation of mechanisms and beneficial effects of EPA and DHA intake found by proteomics and lipidomics tools.

Therefore, proteomics and lipidomics can largely help understand some of the mechanisms behind the beneficial effect of marine lipids against chronic disease induced by diet. These mechanisms are mainly related to competence from enzymes involved in lipid de novo synthesis and oxidation, modulation of anti-inflammatory and antioxidant pathways as well as protein homeostasis. Data also reflected that the effect of dietary marine lipids is closely dependent on their doses, the EPA and DHA ratio, diet components or health status of patients, among others. A schematic representation of mechanisms and beneficial effects of EPA and DHA intake found by proteomics and lipidomics tools is shown in Figure 1.

## 4. Concluding Remarks and Final Considerations

Proteomics and lipidomics approaches constitute valuable tools for studying the effects of marine lipids on metabolic health and disease. In spite of their recent application to nutrition research, these omics have already allowed the identification of numerous metabolic molecules and pathways, tissues and physiological processes which are modulated by the consumption of marine lipids. These findings confirm some of the mechanisms previously suggested by genomics and transcriptomics tools, but they also provide new insights revealing the existence of novel mechanisms and target molecules of great interest for the growing field of food bioactives and personalized nutrition. Proteomics and lipidomics contribute to the discovering of new biomarkers of disease and support the optimal design of both preventive and palliative nutritional strategies against the pathologies in which marine lipids have previously demonstrated their beneficial effects. The progress on the characterization of mechanisms of action of marine lipids at proteomics and lipidomics levels might further identify other diseases in which marine lipids can exert a positive influence. Therefore, although the combination of proteomics and lipidomics approaches is still scarce, it could be the key to understanding the mechanisms involved in the beneficial effects of marine lipids. Such combination will offer a complete overview of cellular process contributing to clarify several controversial facts regarding the in vivo role of marine lipids. 

Finally, it should be noted that the enormous complexity of proteomes and lipidomes together with the scant knowledge available have limited the use of omics in the field of marine lipids. This inconvenience is especially significant for lipidomics due to the lack of information for predicting the number of individual lipid molecules present in an organism. Additionally, it is necessary to mention the high variability of results derived from in vivo experiments as well as other caveats related to high instrumental costs or the need for highly qualified staff. The achievements on higher sensitivity and specificity of the modern MS developments, such as imaging MS or top-down approaches, constitute promising tools which can help solve these problems.

## Figures and Tables

**Table 1:** Research articles found in literature which used proteomics to assay health marine lipid effects.

Reference	Marine Lipids Intervention	Experimental Model	Proteomics Tools	Target Proteome	Main Effects
Camargo et al., 2013 [14]	Acute intake of EPA/DHA (1.4:1)	Human suffering MetS	Quantitative 2-DE-MS/MS	PBMCs	5 proteins regulated from cell signaling and interaction, DNA repair, cellular assembly and organization and cell morphology
Rangel-Zúñiga et al., 2015 [15]	EPA/DHA (1.4:1) for 12 weeks	Human suffering MetS	Quantitative 2-DE-MS/MS	PBMCs	17 proteins regulated from immunological diseases and inflammatory response, oxidative stress, inflammation, endoplasmic reticulum stress and DNA repair
Jiménez-Gómez et al., 2014 [16]	EPA/DHA (1.4:1) for 12 weeks	Human suffering MetS	Quantitative 2-DE-MS/MS	White adipose tissue	3 proteins regulated from glucose metabolism
De Roos et al., 2008 [17]	EPA/DHA (2:1) for 6 weeks	Healthy humans	Quantitative 2-DE-MS/MS	Serum	10 proteins regulated from lipoprotein metabolism and inflammation
Burillo et al., 2012 [18]	0.6 g/d EPA and DHA for 5 weeks	Healthy smokers humans	2-DIGE-MS/MS	HDL	12 proteins regulated related to antioxidant, anti-inflammatory and anti-atherosclerotic properties, regulation of complement activation and acute phase response
Ahmed et al., 2014 [19]	EPA/DHA (1:1) for 4 months	Healthy C57BL/6 mice	Quantitative 2-DE-MS/MS	Liver	11 proteins regulated from lipid, carbohydrate, one-carbon, citric acid cycle and protein metabolisms
Wrzesinski et al., 2013 [20]	EPA/DHA (2:1) for 50 weeks	Wistar rats fed HFHS diet	Quantitative 2-DE-MS/MS	Liver mitochondria	54 proteins regulated from fatty acid and amino acid metabolisms, fatty acid oxidation and oxidative phosphorylation
De Roos et al., 2005 [21]	EPA/DHA (2:1) for 3 weeks	APOE*3 Leiden transgenic mice fed HFC diet	Quantitative 2-DE-MS/MS	Liver	44 proteins regulated from glucose and lipid metabolism, oxidation and aging processes
Méndez et al., 2017 [22]	EPA/DHA (1:1) for 28 weeks	Wistar Kyoto rats fed HFHS diet or STD diet	2-DIGE-MS/MS iTRAQ-nanoLC-MS/MS	Liver	6 proteins regulated in STD diet 31 proteins regulated in HFHS diet from lipogenesis and glycolysis, fatty acid beta-oxidation, insulin signaling, oxidative stress and ameliorating endoplasmic reticulum stress
Kalupahana et al., 2010 [23]	EPA	Cell culture	2-DIGE-MS/MS	3T3-L1 adipocytes	27 proteins regulated from carbohydrate and fatty acid and cell metabolism, response to stress, lipogenesis, cytoskeleton organization and biogenesis
Mavrommatis et al., 2010 [24]	EPA/DHA (1.4:1) or DHA for 2 weeks	apoE knockout mice fed HFC diet	Quantitative 2DE-MS/MS	Liver	35 proteins regulated from of lipoproteins metabolism and oxidative stress; 4 of them different between DHA and fish oil
Johnson et al., 2015 [25]	0.5% EPA or 0.5% DHA for 10 weeks	6- or 24-months C57BL/6 mice	Quantitative untargeted nanoLC-MS/MS	Quadriceps muscle	39 proteins regulated by EPA-treated and 32 proteins regulated by DHA-treated old mice related to anticoagulation, anti-inflammatory, reduced FXR/RXR activation EPA decrease protein carbamylation
Méndez et al., 2013 [27]	EPA:DHA 1:1 or 2:1 or 1:2 for 13 weeks	Wistar Kyoto rats	FTSC-carbonyl protein labeling Quantitative 1DE- and 2DE-MS/MS	Plasma, kidney, skeletal muscle, and liver	6 carbonylated protein targets regulated by 1:1 EPA:DHA in plasma and liver
Jourmard-Cubizolles et al., 2013 [28]	2% DHA for 20 weeks	LDLR^−/−^ mice fed atherosclerotic diet	Quantitative 2DE-MS/MS	Aorta	19 proteins regulated from glucose and lipid metabolisms and oxidative stress 12 identified 4-HNE-proteins

**Table 2:** Main classes of lipid mediators derived from arachidonic acid (ARA), eicosapentaenoic acid (EPA) and docosahexaenoic acid (DHA).

Family	Lipid Mediators from ARA		Lipid Mediators from EPA		Lipid Mediators from DHA	
	Nomenclature	Isomers	Nomenclature	Isomers	Nomenclature	Isomers
Monohydroxys	HETE	3-, 5-, 8-, 9-, 11-, 12-, 15, 18-, 19- and 20HETE	HEPE	5-, 8-, 9-, 11-, 12-, 15 and 18HEPE	HDoHE	4-, 7-, 8-, 10-, 11-, 13-, 14-, 16-, 17- and 20HDoHE
Dihydroxys	DiHET (DiHETrE)	5,6-,8,9-, 11,12- and 14,15 DiHETrE	DiHETE	5,6-, 5,12-, 5,15-, 8,15-, 14,15- and 17,18DiHETE	DiHDPA	10,11-, 14,21- and 19,20DiHDPA
Leukotrienes	LT-_4_	LTA_4_, -B_4_, -C_4_, -D_4_ and –E_4_	LT-_5_	LTA_5_, -B_5_, -C_5_, -D_5_ and -E_5_		
Trihydroxys (lipoxins)	LX-_4_	LXA_4_ and –B_4_	LX-_5_	LXA_5_		
Hydroperoxides	HpETE	5-, 8-, 9-, 11-, 12-, 15-, 19- and 20HpETE	HpEPE	5-, 8-, 9-, 11-, 12-, 15 and 18HpEPE	HpDoHE	4-, 7-, 8-, 10-, 11-, 13-, 14-, 16-, 17- and 20HpDoHE
Epoxides	EET (EpETrE)	5,6-, 8,9-, 11,12- and 14,1EET	EEQ (EpETE)	8,9-, 11,12-, 14,15- and 17,18EEQ	EDP (EpDPA)	7,8-,10,11-, 13,14, 16,17- and 19,20EDP
Thromboxanes	TX-_2_	TXA_2_ and -B_2_	TX-_3_	TXA_3_ and -B_3_		
Prostaglandins	PG-_2_	PGA_2_, -B_2_, -D_2_, -E_2_, -G_2_, -H_2_, -I_2_, -J_2_ and –F_2α_	PG-_3_	PGA_3_, -B_3_, -C_3_, -D3, -E_3_, -I_3_, -H_3_ and –F_3α_		
Isoprostanes	IsoP-_2_	8isoPGJ_2_, -A_2_, -E_2_ and-D_2_	IsoP-_3_	8-, 5-, 11-, 12-, 15- and 18isoPGF_3α_		
Resolvins	8-, 5-, 12 and 15isoPGF_2α_		RvE	RvE_1_, -E_2_ and -E_3_	RvD	RvD_1_, -_2_, -_3_ and -_4_
Neuroprotectins					PD	PD_1_
Maresins					MaR	MaR_2_ (13,14DiHDPA) 7-MaR_1_
Keto-derivatives						
Keto-PG	oxoETE	5-, 8-, 9-, 11-, 12-, 15, 19- and 20 oxoETE				

**Table 3:** Research articles found in literature which used lipidomics to assay health marine lipid effects.

Reference	Marine Lipids Intervention	Experimental Model	Lipidomics Tools	Target Lipidome	Main Effects
Ottestad et al., 2012 [34]	0.7 g/day EPA and 0.9 g/day DHA for 7 weeks	Healthy humans	UPLC-MS	Plasma	Decreased 23 lipids Increased PLs and TGs containing EPA and DHA
Rudkowska et al., 2013 [35]	1.9 g/day EPA and 1.1 g/day DHA for 6 weeks	Healthy humans	MS assay kit	Plasma	Increased glyPCs in unsaturated FA
Nording et al., 2013 [36]	1.9 g/day EPA and 1.5 g/day DHA for 6 weeks	Healthy humans	HPLC-GS-MS SPE-LC-MS/MS	Plasma	Increased incorporation of EPA and DHA into 7 lipid classes High variability in 87 lipid mediators measured
Mas et al., 2012 [37]	4 g fish oil/day (35% EPA and 25% DHA) for 3 weeks	Healthy humans	SPE-LC-MS/MS	Plasma/serum	Measured for first time 17R/SHDHA, RvD_1_, and RvD_2_ concentrations RvD_1_ and RvD_2_ into anti-inflammatory and pro-resolving concentration range
Barden et al., 2014 [38]	4 g fish oil/day (35% EPA and 25% DHA) for 5 days	Healthy humans	SPE-LC-MS/MS	Plasma	Increased RvE1, 18R/S-HEPE, 17R/S-HDHA and 14R/S-HDHA
Keelan et al., 2015 [39]	3.7 g/day (27.7% EPA and 56.% DHA) from 20 pregnancy-week	Healthy pregnant women	GC SPE-LC-MS/MS	Placenta	Increased DHA Increased 18-HEPE and 17-HDHA
Barden et al., 2015 [40]	1.4 g EPA/day and 1 g DHA/day in the form of triglycerides for 3 weeks.	Human suffering metabolic syndrome	SPE-LC-MS/MS	Plasma	Increased E-series resolvins in MetS patients and controls, in which also increased D-series resolvin precursors and 14-HDHA
Schuchardt et al., 2014 [41]	1.14 g/day DHA and 1.56 g/day EPA for 12 weeks	Hyperlipidemic men	SPE-LC-MS/MS	Plasma	Increased EPA-derived lipid mediators Less increased DHA-derived lipid mediators
Polus et al., 2016 [42]	3× (430 mg of DHA and 90–150 mg of EPA)/day for 3 months	Obese women	GC-MS LC-MS/MS	Plasma	Increased pro-resolving DHA derivatives
Lankinen et al., 2009 [43]	Fatty or lean fish for 8 weeks	Coronary heart disease patients	GC-MS UPLC-ESI-MS	Plasma	Decreased 59 bioactive lipid species (ceramides, lysoPCs and DGs) by fatty fish Increased cholesterol esters and specific long-chain TGs by lean fish
Midtbø et al., 2015 [44]	Farmed salmon fed with a reduced ratio of ω-3/ω-6 for 10 weeks	C57BL/6J mice fed western diets	LC-MS/MS	Liver	Increased ARA in PLs Increased ceramides Increased ARA-derived pro-inflammatory mediators Decreased lipid mediators derived from EPA and DHA
Dawczynski et al., 2013 [45]	3 g of EPA and DHA (in 1:1 ratio)/day for 10 weeks	Mildly hypertriacylglycerolemic subjects	LC-MS/MS	Plasma Red blood cells	Increased EPA and DHA levels in plasma and red blood cells Increased plasma EPA-derived mediators (PGE_3,_ and 12-, 15- and 18-HEPE)
Padro et al., 2015 [46]	0.375 EPA and DHA g/day for 28 days	Overweight and moderately hypercholesterolemic subjects	LC-MS/MS	LDL	Increased long-chain polyunsaturated CEs Increased ratio PC36:5/lysoPC16:0
Dasilva et al., 2015 [47]	EPA:DHA 1:1 or 2:1 or 1:2 for 13 weeks	Wistar Kyoto rats	SPE-LC-MS/MS	Plasma	Decreased pro-inflammatory ARA eicosanoids by 1:1 and 2:1 ratios
Dasilva et al., 2016 [48]	EPA:DHA 1:1 or 2:1 or 1:2 for weeks	SHROB rats	SPE-LC-MS/MS	Plasma	Decreased pro-inflammatory ARA eicosanoids by 1:1 and 2:1 ratios
Cipollina et al., 2014 [49]	1 g/day EPA and 0.4 g/day DHA for 4 months	Healthy humans	BME reaction	Blood neutrophils	Increased 7-oxo-DHA and 5-oxo-EPA
Balogun et al., 2013 [50]	EPA:DHA 1:1 for 4 months	C57BL/6 mice	LC-MS	Plasma Liver	Increased EPA containing PCs, LPCs, and CEs Increased free ω-3 PUFAs
Poulsen et al., 2008 [51]	0.5 g DHA or EPA ethyl ester/kg body weight/day 4 months	Sprague–Dawley rats	LC-MS/MS	Bone marrow	Increased EPA and DHA Increased LOX mediators biosynthesized from DHA and EPA (lipoxins, resolving D_1_, resolvin E_1_ and protectin D_1_)
Taltavull et al., 2016 [52]	EPA/DHA (1:1) for 24 weeks	Wistar Kyoto rats fed HFHS diet	GS-MS SPE-LC-MS/MS	Liver	Decreased total ceramides Decreased long chain ceramide 18:1/18:0 Increased very long chain ceramides 18:1/24:0 and 18:1/20:0
Caesar et al., 2016 [53]	Menhaden fish oil (25.2g EPA and 18.2 g DHA/100 g) for 11 weeks	C57BL/6 mice fed HF diet	UPLC-MS	Serum Liver	Interaction with gut microbiota increased hepatic levels of cholesterol and cholesteryl esters by lard but not by fish oil
Kuda et al., 2016 [54]	4.3 mg EPA and 14.7 mg DHA/g diet for 5 weeks	C57BL/6J mice fed obesogenic HF diet	SPE-LC-MS/MS	White adipose tissue	Increased anti-inflammatory lipid mediators (endocannabinoid-related Ndocosahexaenoylethanolamine) and pro-resolving lipid mediator protectin D_1_
Flachs et al., 2011 [55]	46% DHA and 14% EPA for 5 weeks	Mice fed obesogenic MF diet	LC-MS/MS	White adipose tissue	Increased anti-inflammatory lipid mediators (15-deoxy-Δ(12,15)-prostaglandin J_2_ and protectin D_1_) in epididymal fat
González-Périz et al., 2009 [56]	6 g/100 g ω-3 PUFAs for 5 weeks	*ob/ob* mice (B6.VLep/J)	SPE-LC-MS/MS	Liver	Inhibited formation of ω-6 PUFAs derived eicosanoids Induced formation of ω-3 PUFAs derived resolvins and protectins
Kalish et al., 2013 [57]	Parental nutrition with fish oil-based lipid emulsions	C57BL6/J mice high-carbohydrate diet	LC-MS/MS	Liver	Induced production of anti-inflammatory and pro-resolving lipid mediators
González-Périz et al., 2006 [58]	1.37% DHA or 1.37% EPA and DHA for 5 weeks	129S2/SvPasCrl mice fed high saturated fat diets	HPLC-GC/MS	Liver	Increased DHA-derived lipid mediators (17*S*-hydroxy-DHA (17*S*-HDHA) and protectin D_1_ by both supplementations
Aukema et al., 2013 [59]	5% or 10% fish oil for 16 weeks	JCR:LA-cp rats	LC-MS/MS	Kidney	Decreased 5-, 9- 11-, 12- and 15-HETE Decreased endogenous renal levels of 6-keto PGF_1α_, TXB_2_, PGF_2α_ and PGD_2_
Gladine et al., 2014 [60]	DHA (0%, 0.1%, 1% or 2% of energy) for 20 weeks	LDLR^−/−^ mice	GC-MS SPE-LC-MS/MS	Plasma Liver	Increased DHA Increased F4-neuroprostanes (DHA peroxidized metabolites)
Skorve et al., 2015 [61]	Fish oil or krill oil for 6 weeks	C57BL/6 J mice fed HF diet	GC-MS UPLC-MS/MS	Liver Brain	Decreased unsaturated fatty acids by fish and krill oils Decreased ceramides and DGs in liver and brain by krill oil Increased CEs by krill oil in liver Decreased plasmalogens by fish oil in liver Increased hepatic sphingolipids and ARA fatty acid levels more by krill than fish oil in liver Increased ceramides and lactosylceramides more by fish than krill oil in brain
Polus et al., 2015 [62]	EPA	Cell culture	GS-MS LC-MS/MS	Human subcutaneous adipose tissue stromal vascular fraction cells	Decreased pro-inflammatory mediators from ARA Increased anti-inflammatory eicosanoid from EPA
Capel et al., 2015 [63]	DHA	Cell culture	GC-FID LC-MS/MS	C2C12 myotubes	Restoring cellular acylcarnitine profile
Ting et al., 2015 [64]	EPA or DHA	Cell culture	LC-MS/MS	H9c2 cardiac myoblast	Elevation of less unsaturated and ω-3 cardiolipin species mainly by DHA
Lankinen et al., 2011 [65]	Fatty fish and other bioactive compounds for 12 weeks	Metabolic syndrome patients	UPLC-ESI-MS	Plasma	25 altered lipids, including multiple TGs incorporating the long chain ω-3 PUFAs
Wu et al., 2015 [66]	ω-3 PUFA (6.5 g/day) and l-alanyl-l-glutamine (8 g/day) for 3 months	Patients with chronic heart failure	LC-MS	Plasma Skeletal muscle	Increased uptake EPA and DHA Decreased total ceramides and ceramides 22:1 and 20:1
Mas et al., 2016 [67]	ω-3 fatty acids (4 g), Coenzyme Q10 (CoQ) (200 mg) or both for 8 weeks	Patients with chronic kidney disease	LC-MS/MS	Plasma	Increased 8-HEPE, 17-HDHA and RvD1 by ω-3 PUFAs
Bondia-Pons et al., 2014 [68]	0.5% or 1.5% total energy intake EPA and DHA 365 mg or 2900 mg of polyphenols	Patients with metabolic syndrome	UPLC-QTOF-MS	Plasma and HDL fraction	Increased plasma highly unsaturated long-chain TGs and EPA and DHA-containing PLs by ω-3 diets Decreased plasma low unsaturated PLs, PCes, LysoPCs and PCps containing ARA by ω-3 diets Increased PCs and TGs containing DHA or EPA by ω-3 diets in HDL fraction Decreased PCes and PCps containing ARA and medium-chain PCs by ω-3 diets in HDL fraction Decreased PCs and Pes, several alkyl and alkenyl etherlipids containing 16:0 and saturated and low-unsaturated PCs and PEs by both ω-3 and polyphenols diet
Dasilva et al., 2017 [69]	EPA/DHA (1:1) Grape polyphenols for 24 weeks	Wistar Kyoto rats fed HFHS diet or STD diet	GS-MS SPE-LC-MS/MS	Plasma Liver Adipose tissue	Decreased ω-6/ω-3 index in plasma and membranes by ω-3 diets Decreased ARA pro-inflammatory lipid mediators by ω-3 diets Increased desaturases related to EPA and DHA synthesis by ω-3 diets Decreased desaturases related to ARA synthesis by ω-3 diets Combination ω-3&polyphenols cooperative down-regulated Δ5D related with ARA synthesis, decreased COX activity on ARA and total FFA in plasma into STD and HFHS diets

**Table 4:** Research articles found in literature which used both proteomics and lipidomics to assay health marine lipid effects.

Reference	Marine Lipids Intervention	Experimental Model	Proteomics and Lipidomics Tools	Target Proteome and Lipidome	Main Effects
Bakker et al., 2010 [70]	380 mg EPA and 260 mg DHA and other anti-inflammatory compounds for 5 weeks	Healthy overweight men	HumanMAP GS-MS LC-MS/MS	Plasma	Regulated plasma proteins and plasma metabolites (lipids, free fatty acids, and polar compounds) related to modulation of inflammation, improved endothelial function, oxidative stress and increased fatty acid oxidation.
Pellis et al., 2012 [71]	Postprandial response in anti-inflammatory mix-supplemented men Acute intake	Healthy overweight men	HumanMAP GS-MS	Plasma	31 regulated proteins and lipids involved in amino acid metabolism, oxidative stress, inflammation and endocrine metabolism.
López et al., 2015 [72]	ω-6:ω-3 in 442:1 ratio for 5 months	Aging C57BL/6J mice previously suffered myocardial infarction	Protein immunoblot analysis LC-MS/MS	Plasma	Increased VCAM-1, macrophage inflammatory protein-1, D40 and myeloperoxidase Increased ARA and 12(*S*)-HETE and altered levels of inflammation-resolving enzymes 5-LOX, COX-2, and heme oxygenase-1
